# Amelioration of autophagy and inflammatory signaling pathways via α-lipoic acid, burdock and bee pollen versus lipopolysaccharide-induced insulin resistance in murine model

**DOI:** 10.1016/j.heliyon.2023.e15692

**Published:** 2023-04-23

**Authors:** Rehab M. Abdel-Megeed, Mai O. Kadry

**Affiliations:** Therapeutic Chemistry Department, Pharmaceutical and Drug Industries Research Institute, National Research Center, El Buhouth St., Dokki, Cairo, 12622, Egypt

**Keywords:** Lipopolysaccharide, Type 2 diabetes, Insulin resistance, PTEN, STAT5A, CHOP, ATF-4

## Abstract

Lipopolysaccharide (LPS) has previously been implicated in insulin resistance by generating an innate immune response and activating inflammatory cascades. Many studies have discovered a relationship between high levels of serum LPS and the advancement of diabetic microvascular problems, indicating that LPS may play a role in the control of critical signaling pathways connected to insulin resistance. The current study focused on signaling pathways linked to insulin resistance and explored probable mechanisms of LPS-induced insulin resistance in a murine model. It next looked at the effects of burdock, bee pollen, and -lipoic acid on LPS-induced inflammation and autoimmune defects in rats. LPS intoxication was induced via *ip* injection for one week in a dose of 10 mg/kg followed by α-lipoic acid, Burdock and bee pollen in an oral treatment for one month. Following that, biochemical and molecular studies were performed. The RNA expression of the regulating genes STAT5A and PTEN was measured. In addition, ATF-4 and CHOP as autophagy biomarkers were also subjected to mRNA quantification. The results demonstrated a considerable improvement in the -lipoic acid, Burdock, and bee pollen treated groups via modifying oxidative stress indicators as well as molecular ones. Furthermore, glucose concentration in serum and α-amylase were also improved upon treatment with the superiority of α-lipoic acid for modulating all estimated parameters. In conclusion: the results declared in the current study suggested that α-lipoic acid could regulate insulin resistance signaling pathways induced by LPS intoxication.

## Graphical abstract

**Figure:**
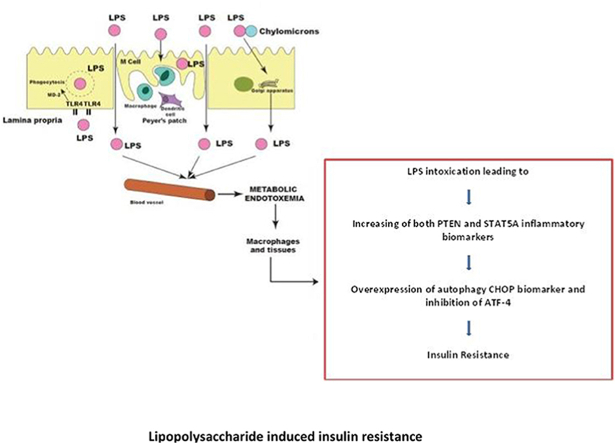

## 1 Introduction

Diabetes mellitus is a chronic metabolic disorder defined by an excessively high level of blood glucose. Insulin deficiency, either absolute or relative, induced by insulin resistance or pancreatic β-cell malfunction are major causes of hyperglycemia [1]. The incidence of diabetes mellitus injured almost 460 million individuals globally and anticipated to reach to 700 million by 2045 [2], which pose a serious risk to world health. The basic pathophysiological processes of diabetes include hyperglycemia, dyslipidemia, insulin resistance, and metabolic pathway dysregulation, which contribute different problems involving various organ functions [1]. With a better knowledge of diabetes, oxidative stress, immunological abnormalities, and genetic mutations, are now recognized as major influence on the development of diabetes and its consequences [1].

Lipopolysaccharides (LPS), commonly known as endotoxins, are a component of the outer membrane of gram-negative bacteria that are released during lysis. LPS is amphiphilic and tripartite in structure, with a highly changeable O antigen, core oligosaccharide, and lipid A (the primary virulence component) [3]. LPS, as a pathogen-associated molecular pattern (PAMP) in gram-negative bacteria, can stimulate innate immune defense and initiate inflammatory cascades in the host [4]. Several investigations involving innate immune defense have established a link between high levels of serum LPS and the advancement of diabetic microvascular problems [5].

The fundamental mechanisms of LPS -induced inflammation involve activating the toll-like receptor 4 (TLR4) on immune cells, which then triggers an intracellular response by NF-kB in the nucleus, followed by the release of chemokines and cytokines. Moreover, LPSs cause the host effector cells to produce a significant number of endogenous pro-inflammatory cytokines by engaging with certain receptors on these cells. These cytokines are produced excessively, which triggers an unregulated inflammatory response that eventually results in severe physiological diseases in the body [6].

Insulin resistance in the myocytes is critical for the evolution of type 2 diabetes mellitus (T2DM). The gut microbiota has recently been connected to this pathogenic process. LPS is suggested to be a causative factor for insulin resistance. Recent investigations have also found higher plasma LPS concentrations in several groups of obese and T2DM patients [7]. Recent investigations have recorded an obvious elevation in LPS values in T2DM plasma samples [8]. Furthermore, a high-fat diet illustrated a significant elevation of LPS levels in both healthy and T2DM individuals; meanwhile LPS administration could rapidly induce insulin resistance [9]. As, the elevated LPS concentration have a major impact on mild acute inflammation, which is a central feature of insulin resistance [10].

Proteins, organelles, lipids, DNA, and RNA are transported to lysosomes via the autophagy route to be broken down and recycled in the vacuole. Autophagy fuels a constant flow of materials in a cell's degradation-regeneration cycle and offers reservoirs of raw materials for anabolic activities [11]. Contrarily, autophagy is thought to determine a cell's fate and can cause type 2 programmed cell death, a sort of non-apoptotic cell death [12]. Therefore, the cellular or environmental context may determine whether autophagy promotes cell death or shields cells from certain forms of damage. Since the bulk of the genes involved in the metabolism and storage of glucose are found in hepatocytes, the liver plays a crucial role in maintaining glucose homeostasis [13]. Insulin resistance conditions increase hepatic glucose production and the ensuing non-suppressible hyperglycemia that causes diabetic hepatopathy [13]. ER stress has been identified as a significant component throughout numerous diabetes etiologies [13,14], which further produces oxidative stress that results in subcellular and macromolecular damages. Most of cellular macromolecules, proteins are sensitive to oxidation by ROS [14] and provide an additional burden on the ER for protein synthesis, folding, and trafficking. Increased oxidative stress results in the breakdown of ER resident proteins and chaperones, which obstructively accumulates misfolded proteins in the ER [15]. Concurrently, phosphorylation of eIF2α promotes translation of ATF4 (activating transcription factor 4) and CHOP (CEBP homologous protein) proteins [16]. At the initiation, ATF4 and CHOP induce autophagy by Promoting the expression of p62, Atg10 (autophagy related 10), Atg5 and Atg7 for protein digestion enhancing ATF4/CHOP-mediated cell death [17].

PTEN plays an important role as a negative regulator of insulin signaling pathways via its function in the dephosphorylation of the phosphoinositide 3-kinase (PI3K) pathway [18]. The control of insulin signaling and glucose homeostasis has been linked to PTEN dysregulation because the PI3K pathway is a significant signaling network that is activated in response to insulin [19]. Insulin binds to the insulin receptor (IR), which causes canonical activation of PI3K, an enzyme that phosphorylates membrane-bound PIP2 to PIP3 and begins the insulin signaling process [20]. As a pathway's negative regulator, PTEN converts PIP3 to PIP2 to suppress the adverse effect of PI3K signaling in response to insulin. Accordingly, insulin resistance, a significant pathogenic mechanism in type 2 diabetes (T2D), can be prevented by increased insulin signaling in several organs as a result of loss-of-function mutations in PTEN [21]. PTEN may therefore be a desirable target for therapeutic intervention in T2D.

α- Lipoic acid which is commonly found in the human diet is absorbed and converted to the dithiol compound intracellularly by NAD (P) H-dependent enzymes. It can scavenge reactive oxygen species, replenish physiological antioxidants, chelate metal ions, and activate insulin signaling besides its crucial role in the energy metabolism in mitochondria [22]. α- Lipoic acid may contribute as an anti-inflammatory agent in addition to improving diabetic neurovascular and metabolic problems [[23], [24], [25]].

Bee pollen was previously recorded to be rich protein, notably free amino acids, as well as carbohydrates, lipids, vitamins, and minerals [26]. Bee pollen also includes trace amounts of flavonoids and phenolic substances [27]. Furthermore, bee pollen has been shown to exhibit antioxidant and radical scavenging capabilities [28]. Anti-inflammatory and antioxidant effects of bee pollen have been previously investigated [29].

Burdock (*Arctium lappa*) is a traditional Chinese medicinal herb that is also edible plant. Burdock seeds and roots are frequently used to treat inflammation-related disorders. The anti-inflammatory efficacy may be due to flavonoids' high anti-inflammatory and antioxidant characteristics, as well as their high free radical scavenging capabilities [[30], [31], [32]].

Therefore, the objective of the present study is to explore the efficacy of Burdock, bee pollen and α-lipoic acid in modulating signaling pathways associated with insulin resistance induced via lipopolysaccharide in rat model due to their anti-inflammatory and antioxidant activities.

## 2 Materials and methods

### 2.1 Chemicals

Lipopolysaccharide and α-Lipoic acid were purchased from Sigma-Aldrich Co (St. Louis, MO, USA). Burdock and bee pollen were obtained from Biolife (USA). Kits for extraction of total RNA and SYBR green RT-PCR kits were provided from Qiagen (Helden, Germany). Primers for PTEN, STAT-5A, ATF-4 and CHOP gene expression were purchased from thermos Fisher Co. (Waltham, AM, USA). Kits for antioxidant determination were obtained from Biodiagnostic Co. (Giza, Egypt). All other chemicals were of the greatest analytical grade.

### 2.2 Animals

In the current study, 48male Wister albino of 200 gm ± 20 were obtained from animal house, National Research Center (NRC). Animals were kept in room temperature and under ordinary light/dark cycle. Furthermore, they were kept in suitable cages (8/each) and freely provided by water pelleted food ration. All animals were kept in the above mentioned conditions for one week prior to procedure starting point for acclimatization. All animals in the current experiment were approved by The Animal Care and Use Committee of NRC's ethical standards and guidelines (24411122022).

### 2.3 Experimental design

To start the experiment, rats were divided into six groups as described below:

Group 1: Negative control healthy animals that were administered saline.

Group 2: Lipopolysaccharide intoxicated rats through *ip* injection for one week (total dose 20 mg/kg) and served untreated [33].

Group 3: Lipopolysaccharide-intoxicated rats then treated via an oral dose of α-Lipoic acid (10 mg/kg daily) for one month [34].

Group 4: Rats treated post lipopolysaccharide intoxication via an oral dose of bee pollen (3 mg/kg daily) for one month [35].

Group 5: Lipopolysaccharide-intoxicated rats then were treated by Burdock in a dose of 5 mg/kg daily for one month [36].

Group 6: Injected lipopolysaccharide animals then given a one-month oral treatment regimen consisting of a mixture of α-lipoic acid, bee pollen and burdock.

### 2.4 Blood sampling

At the end of the experiment, animals were subjected to anesthesia using Co_2_. Blood samples were collected from the retro-orbital vein. Additionally, all sera were separated post centrifugation.

### 2.5 Measured parameters

#### 2.5.1 Determination of serum malondialdehyde

Using assays that are available for purchase and were given by Biodiagnostic Company, malondialdehyde reactivity was assessed (Giza, Egypt) [37].

#### 2.5.2 Glutathione reductase

Glutathione reductase activity was estimated using biodiagnostic kits (Giza, Egypt) [38].

Quantitative Real Time Polymerase Chain Reaction for gene expression.

Total RNA was isolated from blood samples using the Qiamp small kit as directed by the manufacturer (Qiagen; USA). Next, using one-step QuantiTecht SYBR green (Qiagen; USA) kits, RT-PCR analysis was done to determine the mRNA gene expression of PTEN, STAT-5A, ATF-4, and CHOP. Forward and reverse primer sequences were presented in Table 1. Denaturing double-stranded cDNA was used for the quantification process, which next involved annealing genomic DNA to forward and reverse primers at temperatures appropriate for each primer. To assess each gene's relative expression against -actin as a reference gene, a comparative CT (2-ΔΔCT) method was applied [39].

**Table 1:** Primer sequence of PTEN, STAT5A, ATF-4 and CHOP genes (β-actin used as a reference gene).

Gene	Forward sequence	Reverse sequence
PTEN	5′-CATTGCCTGTGTGTGGTGATA-3′	5′-AGGTTTCCTCTGGTCCTGGTA-3′
STAT5A	5'- TTACTGAAGATCAAGCTGGGG 3'	5' TCATTGTACAGAATGTGCCGG3'
ATF-4	5'-AGTGGCATCTGTATGAGCCCA-3'	5'-GCTCCTATTTGGAGAGCCCCT-3'
CHOP	5'-ATGGCAGCTGAGTCATTGCCTTTC-3'	5'-AGAAGCAGGGTCAAGAGTGGTGAA-3'
β- actin	5-CTTTGATGTCACGCACGATTTC-3	5-GGGCCGCTCTAGGCACCAA-3

#### 2.5.3 NMR spectroscopy

The NMR spectra of lipopolysaccharide were recorded on a Jeol 500-MHz (Japan) NMR spectrometer equipped. Samples were dissolved in DMSO. Signals were referenced to internal acetone (H 2.225 ppm, C 30.89 ppm), and the standard of software was used to acquire and process these data [40].

### 2.6 Statistical analysis

The acquired information was examined using SPSS software, version 16. The mean SE was used to express each value. Following a one-way analysis of variance, Turkey's multiple comparisons post hoc test was employed to look at statistically significant differences between groups (ANOVA). When P value less than 0.05, significance was taken into account.

## 3 Results

### 3.1 Oxidative stress modulation

**Fig. 1:**
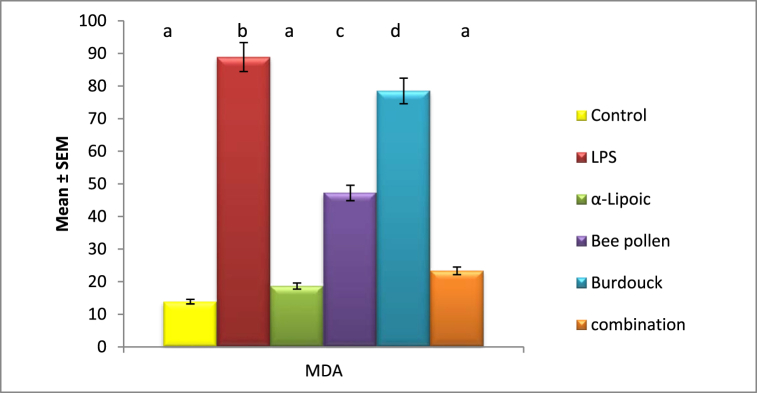
Impact of α-Lipoic acid, Bee pollen and Burdock on MDA 3 against Lipopolysaccharide induced insulin resistance. Data are expressed as mean ± S.E.M (n = 10). p ≤ 0.05 value is considered significant. Groups having the same letter are not significantly different from each other, while those having different letters are significantly different from each other.

**Fig. 2:**
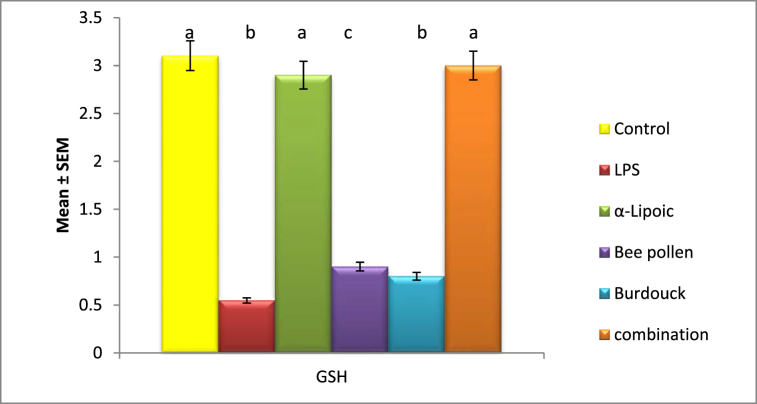
Impact of α-Lipoic acid, Bee pollen and Burdock on GSH against Lipopolysaccharide induced insulin resistance. Data are expressed as mean ± S.E.M (n = 10). p ≤ 0.05 value is considered significant. Groups having the same letter are not significantly different from each other, while those having different letters are significantly different from each other.

Malondialdehyde levels significantly increased in the lipopolysaccharide-impaired groups, reaching 88.9 nmol/ml compared to the negative control group (13.866 nmol/ml). However, glutathione levels significantly decreased, falling to 0.547 mmol/g of tissue from 3.103 mmol/g in healthy animals. Malondialdehyde levels were also shown to be clearly modulated across all treatment regimens, particularly with -Lipoic acid, indicating the most substantial influence (Fig. 1, Fig. 2).

### 3.2 Estimation of glucose level and α-amylase in serum

Lipopolysaccharide intoxication recorded a significant elevation in glucose level recording 169.16 mg/dl as compared to negative control animals (91 mg/dl). Meanwhile, a considerable fall in glucose level was explored after - lipoic acid to be close to normal value (93.1212 mg/dl) demonstrated - lipoic acid's superiority in controlling glucose level in serum sample. Nevertheless, bee pollen therapy and combined treatment groups show no change in glucose levels. Nonetheless, a considerable improvement in glucose level was observed after burdock therapy, but it was smaller than that reported in the -lipoic acid treated group. (Table 2).

**Table 2:** Impact of α-lipoic acid, Burdock and Bee pollen against LPS intoxication on serum glucose level in rats. Groups having different letters are expressed as significant, on the other hand, those have the same letters are considered non-significant. Data are expressed as mean ± (n = 8). P < 0.05

Groups	Glucose concentration (mg/dL)
negative control	91 ± 5.07 ^(a)^
LPS	169.16 ± 4.09 ^(b)^
α- lipoic acid	93.1212 ± 3.92 ^(a)^
Burdock	167.7 ± 2.63 ^(b)^
Bee Pollen	178.46 ± 2.37^(c)^
Combination	168.97 ± 4.89^(b)^

On the other hand, α-amylase concentration recorded a significant reduction (149.79 U/L) as comparing to healthy groups (542.66 U/L). Additionally, - lipoic acid therapy results in a considerable improvement of 444.266 U/L. (Table 3).

**Table 3:** Impact of α-lipoic acid, Burdock and Bee pollen against LPS intoxication on serum α-amylase reactivity in rats. Groups having different letters are expressed as significant, on the other hand, those have the same letters are considered non-significant. Data are expressed as mean ± (n = 8). P < 0.05

groups	α- amylase concentration (U/L)
negative control	542.66 ± 35.45^(a)^
LPS	149.79 ± 17.49^(b)^
α- lipoic acid	444.266 ± 29.39^(c)^
Burdock	197.33 ± 21.45^(d)^
Bee Pollen	394.76 ± 37.25^(e)^
Combination	246.00 ± 12.45^(f)^

### 3.3 Modulation of PTEN and STAT-5A gene expression

As compared to the negative control animal, lipopolysaccharide intoxication increased PTEN and STAT-5A gene expression by 1.7 and 5 fold changes, respectively. Meanwhile, a significant reduction in PTEN as well as STAT-5A was investigated upon α-lipoic acid to be close to normal value (1 and 0.8 fold changes) reflecting the superiority of α-lipoic acid in modulating PTEN and STAT-5A gene expression. Treatment with bee pollen, on the other hand, shows no improvement in PTEN gene expression. Nonetheless, a considerable modification in STAT-5A gene expression after bee pollen administration was found to be around normal value.

**Fig. 3:**
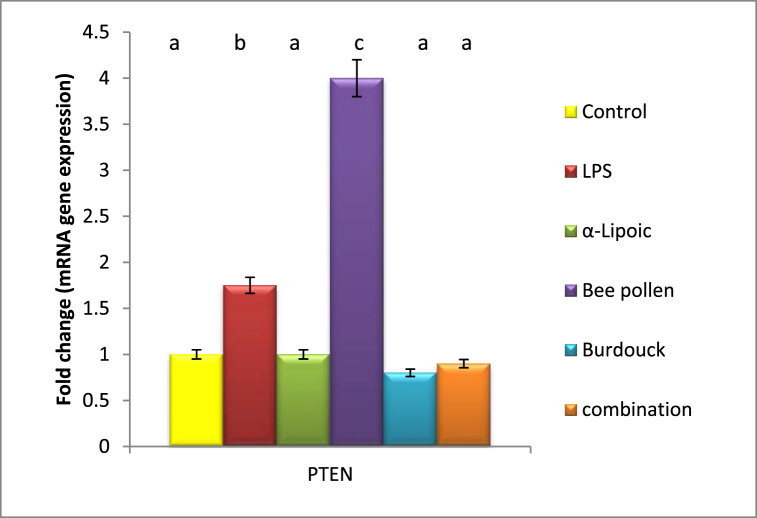
Impact of α-Lipoic acid, Bee pollen and Burdock on PTEN gene expression against Lipopolysaccharide induced insulin resistance (β-actin was used as reference gene). Data are expressed as mean ± S.E.M (n = 10). p ≤ 0.05 value is considered significant. Groups having the same letter are not significantly different from each other, while those having different letters are significantly different from each other.

**Fig. 4:**
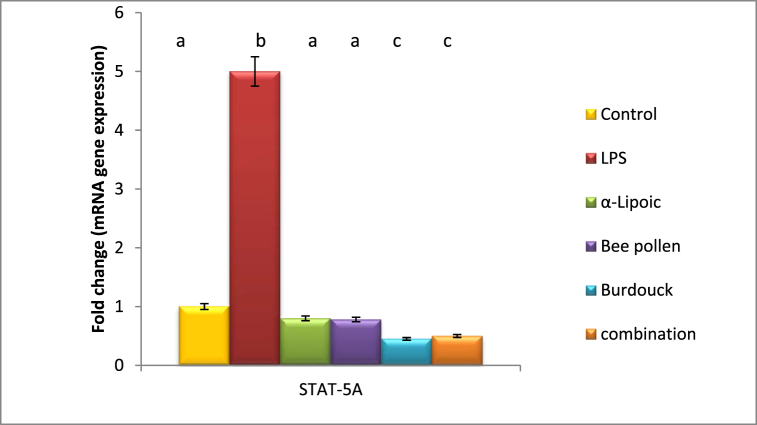
Impact of α-Lipoic acid, Bee pollen and Burdock on STAT-5A gene expression against Lipopolysaccharide induced insulin resistance (β-actin was used as reference gene). Data are expressed as mean ± S.E.M (n = 10). p ≤ 0.05 value is considered significant. Groups having the same letter are not significantly different from each other, while those having different letters are significantly different from each other.

Burdock treatment as well as combination of all treatment regimens notably declared downregulation in the expression of PTEN and STAT-5A genes (Fig. 3, Fig. 4).

### 3.4 Regulation of autophagy signaling pathway

**Fig. 5:**
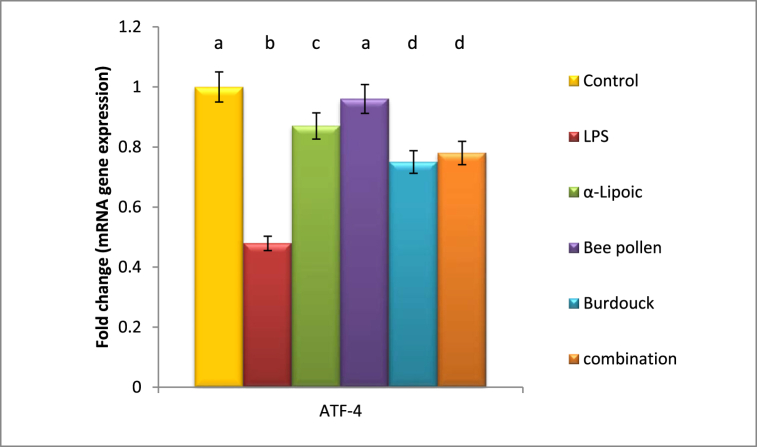
Impact of α-Lipoic acid, Bee pollen and Burdock on ATF-4 gene expression against Lipopolysaccharide induced insulin resistance (β-actin was used as reference gene). Data are expressed as mean ± S.E.M (n = 10). p ≤ 0.05 value is considered significant. Groups having the same letter are not significantly different from each other, while those having different letters are significantly different from each other.

**Fig. 6:**
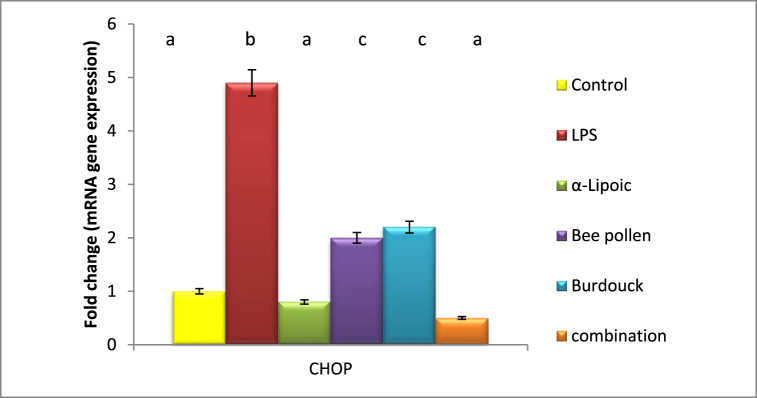
Impact of α-Lipoic acid, Bee pollen and Burdock on CHOP gene expression against Lipopolysaccharide induced insulin resistance (β-actin was used as reference gene). Data are expressed as mean ± S.E.M (n = 10). p ≤ 0.05 value is considered significant. Groups having the same letter are not significantly different from each other, while those having different letters are significantly different from each other.

Data in the current study declared a significant reduction in ATF-4 gene expression post lipopolysaccharide intoxication recording 0.479 fold changes as compared to healthy group. On the other, a significant elevation was recorded in the expression of CHOP gene with 4.9 fold change as compared to negative control group. All of the above-mentioned regimens resulted in an apparent modification of ATF-4 gene expression near normal levels, with the superiority of the bee pollen-treated group recoding a 0.96 fold change. Additionally, a substantial downregulation was seen following treatment with -lipoic acid, bee pollen, Burdocks, and a combination of all treated groups, with the superiority of α-lipoic acid treatment group recording 0.8 fold changes as compared to negative control group. (Fig. 5, Fig. 6).

### 3.5 NMR spectroscopy of LPS

**Fig. 7:**
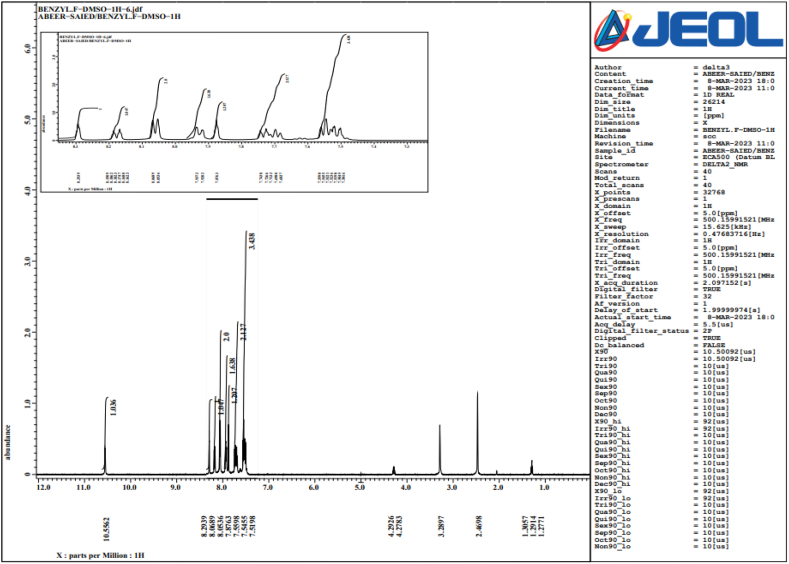
NMR for LPS dissolved in Benzyl DMSO.

**Fig. 8:**
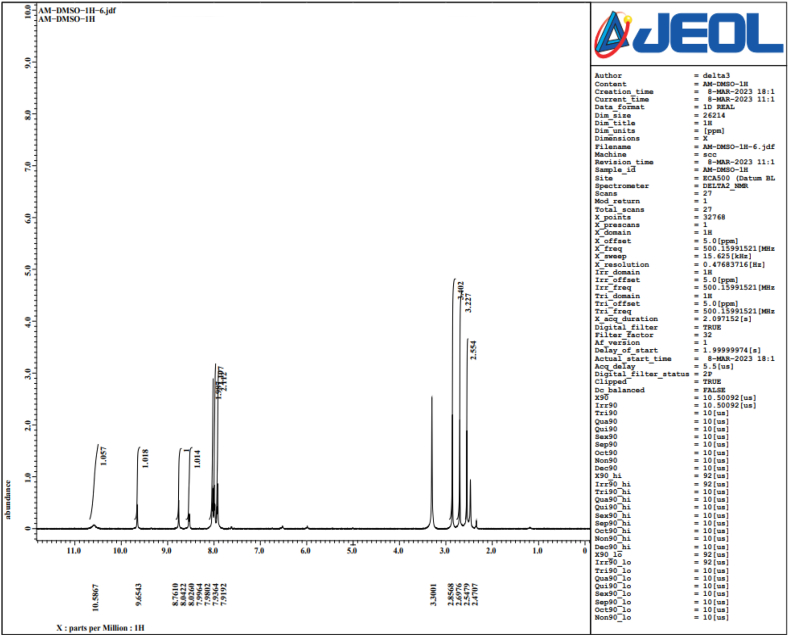
NMR for LPS dissolved in AM- DMSO.

**Fig. 9:**
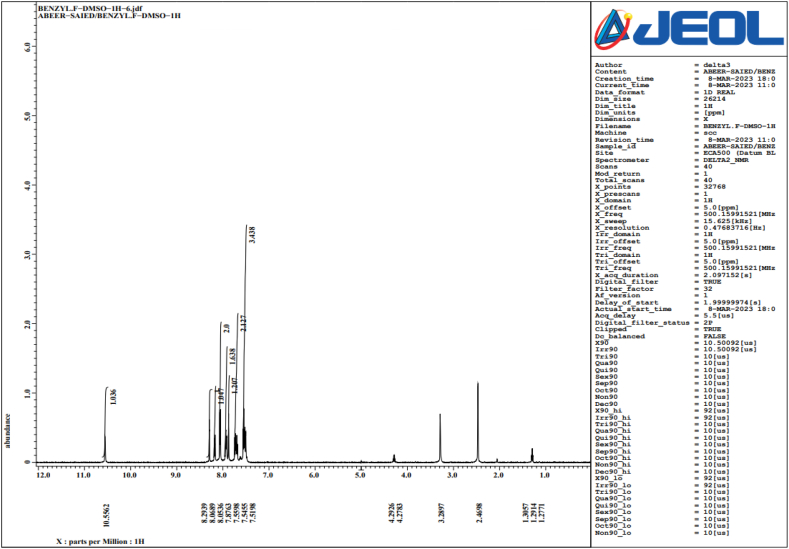
NMR for LPS dissolved in Benzyl DMSO.

Complex organic solvent solutions, such DMSO, were able to produce well-resolved NMR signals when LPS, which still contains the hydrophobic lipid A moiety, was exposed to NMR analysis. The inner core oligosaccharide and other parts, such lipid A, are frequently thought to contribute to the endotoxicity of LPS, which is usually believed to be the endotoxic moiety of the entire LPS molecule (Fig. 7, Fig. 8, Fig. 9).

## 4 Discussion

Insulin resistance is the most common risk factor for metabolic syndrome. As it reduces the response of target cells to insulin owing to a decline in their sensitivity to insulin secretion, insulin resistance has a negative effect on the overall metabolism [41]. Characteristic outcomes caused by insulin resistance include metabolic disorders, such as type 2 diabetes, obesity and glucose intolerance. Insulin resistance is also recognized as a cardiovascular disease related factor and is characterized by endothelial dysfunction [42].

On the other hand, LPS from gut microbiota was reported as linker between inflammation and insulin resistance-induced metabolic syndrome [10]. However, whether LPS directly regulated signaling pathways associated with insulin resistance, the related molecular mechanisms were less studied. Herein we reported that LPS elevated oxidative stress biomarkers as well as mRNA gene expression of both STAT-A5 and PTEN in addition to a notably increase of autophagy signaling pathways ATF-4 and CHOP.

It was reported that the LPS could possibly induce oxidative stress in mice as well in rats due to the tissue damage which increased the production of pro-inflammatory cytokines and generation of ROS. The capability of LPS for induction of oxidative stress was previously studied in murine model [43].

One of the various effects of oxidative stress is peroxidation of unsaturated fatty acids in membrane. The degree of lipid peroxidation, measured as MDA, was used to explain the role of LPS in peroxidation of lipids. The concentration of MDA was increased immediately upon LPS administration [44]. Different previous studies investigated significant elevation of MDA levels upon LPS intoxication [45]. Furthermore, lipid peroxidation levels were elevated upon high doses of LPS in Fischer rats [46]. Also, LPS (200 μg/mouse, for 2 h) induced lipid peroxidation increment [47]. In the current study, animals treated byα-lipoic acid only as well as combination treated group declared a significant modulation of oxidative stress. Previously, it was documented that α-lipoic acid plays an important role in modulating the adverse effect of LPS-induced oxidative stress that may develop multiple organ failure [48,49].

Furthermore, as previously recorded, diabetes is considered as an autoimmune disease as well as metabolic one. 3. Among diabetic subjects, suggesting that LPS induced inflammatory responses could accelerate different metabolic disorders including diabetes. Data recorded form the current study declared a significant elevation of serum blood glucose level upon LPS intoxication.

Consistent with our findings, previous study investigated that macrophages may respond to LPS challenge in a more exaggerated manner under hyperglycemic conditions than under normal glucose conditions, and noticed hyperglycemia via activation of signal transduction and subsequent cytokine production in LPS stimulated monocytic cells [50].

Our study declared a significant reduction in glucose level upon α-lipoic acid treatment. Improvement of glucose levels by α-lipoic acid has been previously recorded in T2DM cases through improving the diabetes-related deficit in glucose metabolism in addition to activation of insulin signaling pathways [51]. Furthermore, their finding declared the possible correlation between oxidative stress induced by LPS and insulin resistance [52].

On the other hand, burdock treatment as well as bee pollen doesn’t declare any improvement in glucose. These results are in contrast to the previous in vitro study investigation which proved the efficacy of burdock in lowering glucose concentration [53].

Herein, a significant reduction was noticed in the expression of PTEN upon LPS administration. Furthermore, a significant improvement was declared upon α-lipoic acid, burdock as well as combination treatment. On the other hand, Bee pollen didn’t declare remarkable modulation in PTEN gene expression.

Conversely, studies have reported that polymorphisms in PTEN are associated with increased insulin resistance or metabolic syndrome, a major risk for the onset of T2D. For example, substitution of cytosine to guanine in position 9 in the 50-untranslated region in PTEN is commonly found in Japanese individuals with T2D. Indeed, individuals with this polymorphism have increased PTEN protein levels, decreased AKT phosphorylation, and increased insulin resistance when compared with healthy controls [54]. Furthermore, metabolic syndrome individuals declared a reduction in PTEN promoter supporting that PTEN down-regulation has a protective effect in T2D [55]. The PTEN knockout (KO) mice models have been the most useful experimental tools in elucidating the metabolic activities of PTEN in vivo. Mice with PTEN loss only in metabolic tissues, as opposed to mice with whole-body PTEN KO, are alive and enable for the examination of the tissue-specific contributions of PTEN signaling in affecting insulin action and glucose homeostasis [55]. The most of mice with fibroblast KO for PTEN in metabolic organs recorded improvement in metabolic functions, including greater glucose tolerance and insulin sensitivity [56,57].

PTEN as a tumor suppressor is involved in the proliferation of various cells [58]. The reduction of PTEN expression leads to the activation of the PI3K/Akt signaling pathway [59]. Previous study recorded that LPS-induced inhibition of PTEN is directly related to fibroblast proliferation, differentiation and collagen secretion by modulating PI3K/Akt/GSK3β pathway. Furthermore, PTEN expression was previously reduced in vitro upon LPS intoxication; the underlying mechanism may be correlated with LPS-induced activation of some transcription factors as NF-κB and c-Jun [60].

The current finding declared a significant downregulation in groups treated by burdock as well as combination group. This modulation may be caused by a burdock active ingredient that has numerous pharmacological properties and has been shown to have anti-tumor, neuroprotective, antiviral, anti-inflammatory, anti-oxidant, and endoplasmic reticulum (ER) stress regulating actions through regulating NFkB, PI3K/AKT, and Stat pathways [[61], [62], [63]].

Furthermore, a remarkable overexpression in STAT5A gene was demonstrated upon LPS intoxication. Meanwhile an obvious downregulation was declared upon treatment with all treated regimens (α-Lipoic acid, Bee pollen and Burdock). Previous findings suggested that STAT5A participating in suppression of cytokine signaling pathways via STAT gene expression regulation [64].

Autophagy signaling pathways of both ATF-4 and CHOP recorded a significant reduction in their expression upon LPS intoxication. Consistent with our findings, it was demonstrated that hyperglycemia triggered ATF–CHOP signaling pathways upon LPS intoxication that it may promote pro-inflammatory response in macrophages [65].

Furthermore, NMR spectroscopy analysis elucidated the 4-phosphate that is sterically nearby can be replaced by the negatively charged carboxyl group, which is next to the lipid A backbone. In prior research, interactions of intact LPS with natural pathogen-related receptors (CD14, MD-2 and TLR4) in almost natural settings were examined. Conformational characteristics of these interactions revealed knowledge of different biological roles, including insulin resistance.

## 5 Conclusion

In conclusion, the current study confirmed that LPS intoxication is associated to dysregulation of both inflammatory and autophagy signaling pathways which leading to insulin resistance and in hence causing a notable elevation in glucose blood level. The impact of α-Lipoic acid, Bee pollen and Burdock on modulation of all dysregulated biochemical parameters was investigated in the current study. In addition, data in our findings declared the superiority of α-Lipoic acid in modulating all dysregulated parameters. Therefore, it could be concluded that the administration of α-Lipoic acid may be a promising regimen for modulating oxidative stress as well as insulin resistance induced by LPS intoxication [40].

## Author contribution statement

**Rehab Abdel-Megeed**: Conceived and designed the experiments; Performed the experiments; Wrote the paper.

**Mai O. Kadry**: Performed the experiments; Analyzed and interpreted the data; Contributed reagents, materials, analysis tools or data.

## Data availability statement

Data included in article/supplementary material/referenced in article.
